# *Schisandra chinensis* bee pollen’s chemical profiles and protective effect against H_2_O_2_-induced apoptosis in H9c2 cardiomyocytes

**DOI:** 10.1186/s12906-020-03069-1

**Published:** 2020-09-10

**Authors:** Peiying Shi, Qianqian Geng, Lifu Chen, Tianyu Du, Yan Lin, Rongcai Lai, Fei Meng, Zhenhong Wu, Xiaoqing Miao, Hong Yao

**Affiliations:** 1 Department of Traditional Chinese Medicine Resource and Bee Products, College of Animal Sciences (College of Bee Science), Fujian Agriculture and Forestry University, Fuzhou, China; 2 State and Local Joint Engineering Laboratory of Natural Biotoxins, Fujian Agriculture and Forestry University, Fuzhou, China; 3 College of Food Science, Fujian Agriculture and Forestry University, Fuzhou, China; 4 Department of Pharmaceutical Analysis, School of Pharmacy, Fujian Medical University, 1 Xue Yuan Road, University Town, Fuzhou, 350122 People’s Republic of China

**Keywords:** *Schisandra chinensis* bee pollen extract, Nucleosides, Quinic acid nitrogen-containing derivatives, H9c2 cardiomyocytes, H_2_O_2_, Protective effect

## Abstract

**Background:**

*Schisandra chinensis* (Turcz.) Baill bee pollen extract (SCBPE) is often used as a functional food in China due to its good antioxidant property. However, its chemical compositions and effects on H9c2 cardiomyocytes against H_2_O_2_-induced cell injury still lacks of reports thus far. This study aimed to characterize the main components of SCBPE and investigate its protective effects against H_2_O_2_-induced H9c2 cardiomyocyte injury.

**Methods:**

The main components of SCBPE were analyzed via ultraperformance liquid chromatography–quadrupole time-of-flight tandem mass spectrometry (UPLC–QTOF MS/MS). The three main nucleosides in SCBPE were quantitatively analyzed via ultraperformance liquid chromatography–diode array detection. Furthermore, the potential mechanism by which SCBPE exerts protective effects against H_2_O_2_-induced H9c2 cardiomyocyte injury was explored for the first time via cell survival rate measurements; cell morphological observation; myocardial superoxide dismutase (SOD) activity and malondialdehyde (MDA) and glutathione (GSH) level determination; flow cytometry; and quantitative polymerase chain reaction.

**Results:**

Two carbohydrates, three nucleosides, and nine quinic acid nitrogen-containing derivatives in SCBPE were identified or tentatively characterized via UPLC–QTOF MS/MS. The nine quinic acid nitrogen-containing derivatives were first reported in bee pollen. The contents of uridine, guanosine, and adenosine were 2.4945 ± 0.0185, 0.1896 ± 0.0049, and 1.8418 ± 0.0157 μg/mg, respectively. Results of in vitro experiments showed that cell survival rate, myocardial SOD activity, and GSH level significantly increased and myocardial MDA level significantly decreased in SCBPE groups compared with those in H_2_O_2_ group. Cell morphology in SCBPE groups also markedly improved compared with that in H_2_O_2_ group. Results indicated that SCBPE protected H9c2 cardiomyocytes from H_2_O_2_-induced apoptosis by downregulating the mRNA expressions of Bax, cytochrome C, and caspase-3 and upregulating the Bcl-2 mRNA expression.

**Conclusions:**

This study is the first to report that SCBPE could protect against oxidative stress injury and apoptosis in H_2_O_2_-injured H9c2 cells. Results indicated that the nucleosides and quinic acid nitrogen-containing derivatives could be the main substances that exert protective effects against H_2_O_2_-induced H9c2 cardiomyocyte injury.

## Background

Myocardial ischemia leads to characteristic patterns of metabolic and ultrastructural changes that result in irreversible injury [1]. Patients with acute myocardial ischemic syndromes may represent a continuum of diseases ranging from unstable angina to acute infarction [2], and myocardial infarction is a major cause of death and disability worldwide [3]. Oxidative stress and generation of reactive oxygen species (ROS) can contribute to cardiac ischemic injuries [4, 5]. Excess ROS can induce oxidative modification of cellular macromolecules, inhibit protein functions, and promote cell death [6]. Hydrogen peroxide (H_2_O_2_), which belongs to O_2_-derived nonradical species [6], is a main component of ROS and has been widely used as an inducer of oxidative damage in in vitro models [7]. Various natural products from medicinal plants, such as new depsides from the roots of *Salvia miltiorrhiza* [8], *Dendrobium officinale* polysaccharides [9], chlorogenic acid analogs from *Gynura nepalensis* [10], and bioactive constituents from Chinese propolis [11], have been proved to protect H9c2 cardiomyocytes from H_2_O_2_-induced oxidative injury.

Bee pollen, which is used as food for all the developmental stages in the hive, is obtained from the agglutination of flower pollens with nectar and salivary substances of the honeybees [12]. Bee pollen extract can be regarded as a promising therapeutic and nutritional natural food supplement because of its certain functions, such as antioxidative, antimicrobial, anti-inflammatory, hepatoprotective, anticancer immunostimulating, and local analgesic effects [13]. These functional biological properties could be due to the high content of flavonoids and polyphenols and considerable radical scavenging capacity [14]. The bee pollen of *Schisandra chinensis* (Turcz.) Baill, which is often used as a functional food in China, has been found to possess a strong antioxidant activity [15–17]. Our recent study revealed the antioxidative and cardioprotective effects of *S. chinensis* bee pollen extract (SCBPE) on isoprenaline-induced myocardial infarction in rats [18]. However, its protective effects against H_2_O_2_-induced H9c2 cardiomyocyte injury have not been reported yet. This property can also provide scientific evidence for using SCBPE as a functional food for the prevention of myocardial ischemia. With the exception of uridine, which has been isolated from *S. chinensis* bee pollen [18], the other chemical components of *S. chinensis* bee pollen remain unknown. Identification of these components is beneficial to the further development and utilization of functional foods from *S. chinensis* bee pollen.

High-performance liquid chromatography combined with high-resolution tandem mass spectrometry is a powerful tool for determining the structures of interesting compounds because of its complementary capacity to provide accurate molecular weights and structural information. Numerous medicinal and edible plants have been tested via this technique to disclose their chemical components [19–22]. Ultraperformance liquid chromatography (UPLC) can offer high linear velocities and ultrahigh resolution within a short period of time with little organic solvent consumption. UPLC is especially suitable to the analysis of multiple components in edible and medicinal plants [23, 24]. For example, UPLC methods have been widely adopted for the determination of functional ingredients in chilies [24], apples [25], and oranges [26]. Therefore, UPLC can also be a powerful tool for the quantitative analysis of the interesting ingredients in bee pollen.

In this study, the main components of SCBPE were analyzed via UPLC–quadrupole time-of-flight tandem mass spectrometry (UPLC–QTOF MS/MS). The three main nucleoside components in SCBPE were quantitatively analyzed via UPLC-diode array detection (UPLC–DAD). Furthermore, the potential mechanism by which SCBPE exerts protective effects against H_2_O_2_-induced H9c2 cardiomyocyte injury was investigated for the first time via cell survival rate measurements; cell morphological observation; myocardial superoxide dismutase (SOD) activity and malondialdehyde (MDA) and glutathione (GSH) level determination; flow cytometry; and quantitative polymerase chain reaction (qPCR).

## Methods

### Materials and reagents

*S. chinensis* bee pollen was purchased from an apiary in Xuchang, Henan, China in October, 2015, and stored in 4 °C. The voucher specimens (no. 151001), identified by Prof. Xiaoqing Miao based on China national standard GB/T 30359–2013, are stored in the traditional Chinese medicine pharmacology laboratory of Fujian Agriculture and Forestry University. Methanol (HPLC-grade) was purchased from Merck (Darmstadt, Germany). Ultra pure water was provided by China Resources C’estbon Beverage (China) Co., Ltd. (Shenzhen, Guangdong, China). Formic acid (HPLC-grade), ethanol and sucrose were obtained from Sinopharm Chemical Reagent Co., Ltd. (Shanghai, China). Uridine, guanosine and adenosine were provided by Solarbio (Beijing, China). The purities of all the standard compounds were determined to be above 98% by HPLC analysis.

Fetal bovine serum (FBS) and Penicillin/Streptomycin (P/S) were purchased from Gibco (USA). Trypsin and Dulbecco’s modified Eagle medium (DMEM, high glucose) were purchased from HyClone (USA). 3-(4,5-Dimethylthiazol-2-yl)-2,5-diphenyltetrazolium bromide (MTT) was obtained from Biosharp (China), and 0.4% trypan blue was provided by Solarbio (Beijing, China). The assay kits for MDA, GSH, total SOD, total protein, cell apoptosis (Annexin V-fluorescein isothiocyanate (FITC)/propidium iodide (PI)) were provided by Nanjing Jiancheng Bioengineering Institute (Nanjing, China). Vitamin C (Vc) with the purity of 98% was purchased from Sigma-Aldrich Co. (St. Louis, MO, USA). 30% H_2_O_2_ solution, dimethylsulfoxide (DMSO), 0.01 M phosphate buffer solution (PBS, pH 7.2–7.4) and other reagents were purchased from Sinopharm Chemical Reagent Co., Ltd. (Shanghai, China).

### SCBPE sample preparation

Dry *S. chinensis* bee pollen samples were pulverized into powder, and extracted twice by shaking using a shaker with 70% ethanol at a ratio of 1:15 (w/v) at room temperature for 24 h. After filtration, the extract was centrifuged for 10 min at 7500 g, 4 °C. Then the supernatant was concentrated using a rotary evaporator at 40 °C until the ethanol had been removed. Ultimately, the SCBPE was obtained after lyophilization in a freeze dryer.

For UPLC–QTOF MS/MS and UPLC–DAD analysis, 10 mg of SCBPE sample was accurately weighed and dissolved in 1 mL of 70% aqueous methanol. After vortex for 2 min, the solution was centrifuged at 12704 g for 10 min and 1 μL of supernatant was injected into the UPLC. For the in vitro cell experiment, 100 mg/mL of SCBPE stock solution was prepared using DMSO.

### Identification of the main components of SCBPE via UPLC–QTOF MS/MS

UPLC analysis was performed on an Agilent 1290 Infinity LC instrument (Agilent, Waldbronn, Germany) consisting of a quaternary pump, an auto-sampler, a column compartment and a diode-array detector. The samples were separated on a Zorbax SB-C_18_ column (3.5 μm, 100 mm × 2.1 mm i.d.). The mobile phase was a stepwise gradient of 0.1% formic acid aqueous solution (A) and methanol (B) as follows: 10% B at 0–3 min; 10–65% B at 3–10 min; 65–80% B at 10–10.5 min and maintained at 80% B from 10.5 min to 15 min. The UV wavelength was set at 254 nm. The column temperature and the flow rate were set at 20 °C and 0.2 mL/min, respectively.

The UPLC system was connected to an Agilent 6530 QTOF mass spectrometer (Santa Clara, CA, USA) equipped with a dual electrospray ionization (ESI) interface using the following operation parameters: capillary voltage, 3.5 kV in (−)ESI mode or 4.0 kV in (+)ESI mode; nebuliser, 40 psig; drying gas (nitrogen) flow rate, 10.0 L/min; gas temperature, 325 °C; fragmentor, 175 V; skimmer voltage, 65 V; OCT 1 RF Vpp, 750 V. Mass spectra were recorded across the range *m/z* 100–1500. The MS/MS collision energy was set at 20–30 V.

### Quantitation of three nucleosides from SCBPE via UPLC–DAD

The chromatographic conditions of UPLC–DAD were consistent with those of UPLC–QTOF MS/MS except that the mobile phase has been changed to water (A) and methanol (B).

The stock solution was prepared by dissolving uridine (1.00 mg), guanosine (1.05 mg) and adenosine (1.07 mg) with 70% aqueous methanol in the same one volume flask (2 mL). Then one working solution containing 200 μg/mL of uridine, 210 μg/mL of guanosine and 214 μg/mL of adenosine, which was obtained by diluting the stock solution with 70% aqueous methanol, was further diluted with the same solvent to obtain 9 different concentration levels including 1, 1/2, 1/4, 1/8, 1/16, 1/32, 1/64, 1/128, and 1/256 times of the original concentration to obtain 0.78, 1.56, 3.13, 6.25, 12.5, 25, 50, 100, 200 μg/mL of uridine, 0.82, 1.64, 3.28, 6.56, 13.13, 26.25, 52.5, 105, 210 μg/mL of guanosine, and 0.84, 1.67, 3.34, 6.69, 13.38, 26.75, 53.5, 107, 214 μg/mL of adenosine, respectively. All the solutions were stored at 4 °C before the test. The calibration curve for each ingredient was plotted by using the peak area (*y*) versus concentration (*x*). The limit of detection (LOD) for each component was determined at a signal-to-noise ratio of 3, while the limit of quantification (LOQ) was evaluated at a signal-to-noise ratio of 10. The precision was tested by assaying one concentration of mixed standard solution (100 μg/mL of uridine, 105 μg/mL of guanosine and 107 μg/mL of adenosine) within 1 day in six times, which was measured by relative standard deviation (RSD). To evaluate the repeatability, six SCBPE samples were treated and analyzed with the established method. The RSD was taken as the measure of repeatability. The stability of SCBPE sample solutions was analyzed using the established method at 0, 2, 4, 8, 10, 24, 36, and 48 h, respectively. 10 mg of six SCBPE samples was respectively weighed and spiked with known amounts of reference compounds, then prepared as described in the Section of SCBPE Sample Preparation and analyzed with the developed UPLC method. The quantity of each compound was subsequently calculated from the corresponding calibration curve. Recovery (%) was calculated by the equation (amount determined - amount original)/amount spiked × 100%.

### Cell culture

Rat-derived H9c2 cardiomyocytes were purchased from Shanghai FuHeng Cell Center (Shanghai, China). Cells were cultured in DMEM (high glucose) supplemented with 10% fetal bovine serum, 1% penicillin and streptomycin at 37 °C in 5% CO_2_. Cells were passaged and subcultured to 70–80% confluence with 0.25% trypsin (w/v) every 2–4 days.

### Cell viability assay and screening of the appropriate concentration and administration time of SCBPE

H9c2 cells were collected with 0.25% trypsin and re-suspended and then seeded in 96-well multiplates with a final density of 5 × 10^3^ cells per well. After incubation for 24 h, cardiomyocytes were pretreated with 6.25, 12.5, 25, 50, 100, 250, 500 μg/mL of SCBPE for 72 h in order to check the effect of SCBPE on H9c2 cardiomyocytes. Besides, cardiomyocytes were pretreated with 6.25, 12.5, 25, 50, 100, 250, 500 μg/mL of SCBPE and 40 μM of Vc for 24 h, 48 h and 72 h, respectively, followed by exposure to 400 μM of H_2_O_2_ for 2 h. And cardiomyocytes were pretreated with uridine (0.78–100 μg/mL), guanosine (0.2–100 μg/mL), adenosine (0.2–100 μg/mL) and quinic acid nitrogen-containing derivatives prepared in our laboratory (0.2–25 μg/mL) for 48 h and 72 h, respectively, followed by exposure to 400 μM of H_2_O_2_ for 2 h. Then the MTT assay was performed to detect the cell viability as described previously [9]. Briefly, 10 μl of 5 mg/mL of MTT solution was added to each well, and the final concentration of MTT was 0.5 mg/mL. After incubation for 4 h under standard condition, 100 μl of DMSO was added into every well to dissolve the formanzan crystals after that the cell supernatants were removed. And the optical density (OD) was measured at 492 nm using a microplate reader Infinite F50 (Tecan, Männedorf, Switzerland). The results of cell viability were expressed as percentages of control.

### Morphological changes in myocardial cells

H9c2 cells were collected, re-suspended and seeded in 24-well multiplates for culture, and the morphological changes of the myocardial cells in different groups were observed under the inverted microscope (TS-100F, Nikon).

### Determination of SOD activity and MDA and GSH levels

The SOD activity in cultured supernatant and intracellular MDA and GSH levels were determined using commercial kits based on colourimetric methods. After treatment, H9c2 cells in different groups were harvested by centrifugation and the supernatants were used to detect SOD activity. The remaining cells were washed with PBS (pH 7.2–7.4) three times, and then the precipitate obtained through centrifugation was added with PBS (pH 7.2–7.4) and crushed in ice water bath with a manual glass homogenizer. The resulting suspension was collected to analyze the contents of GSH and MDA according to the manufacturer’s instructions.

### Flow cytometric detection of apoptosis

Apoptosis was detected using an Annexin V-FITC kit according to the manufacturer’s recommendations on flow cytometric analysis. After treatment, the H9c2 cells were harvested with non-EDTA trypsin, washed with PBS, and resuspended in binding buffer prior to the addition of Annexin V-FITC and PI. The mixture was incubated for 10 min in the dark at room temperature. Subsequently, cellular fluorescence was measured by flow cytometric analysis using BD Accuri C6 flow cytometer with the C6 Software (BD Biosciences, San Jose, CA, USA).

### QPCR

The mRNA expression of caspase-3, cytochrome C, Bax and Bcl-2 in H9c2 myocardial cells was quantified using real time qPCR. Firstly, cells were routinely collected to extract and isolate the total RNA for each treatment group by using the TRIzol reagent (Invitrogen, Carlsbad, CA, USA) in the light of the manufacturer’s instruction. Next, the quality of the total RNA isolated (including the content and integrity) were analyzed with spectrophotometry and agarose gel electrophoresis, followed by preparing the cDNA with a commercial kit (*TransScript*® One-Step gDNA Removal and cDNA Synthesis SuperMix; Transgen, China) in line with the production’s instructions. QPCR was ultimately carried out on the instrument CFX384 Touch (BIO-RAD laboratories, Inc) with TB Green Premix EX Taq™ II (Tli RNaseH Plus) Kit (Takara, China). GAPDH was used as a housekeeping gene for normalization. The experimental conditions were according to our previous report [27] with a little modification. In brief, the final volume for amplification is 10 μL including 1 μL of cDNA template, 0.4 μL of forward primer, 0.4 μL of reverse primer, 5 μL of TB Green premix EX Taq™ II and 3.2 μL of nuclease-free water. For qPCR, the initial denaturation was fulfilled for 3 min at 95 °C, followed by 40 cycles annealing for 5 s at 95 °C and 30 s at 58 °C with subsequent melting curve analysis, and then increasing the temperature from 65 °C to 95 °C with a rate of 0.5 °C per 5 s. The mRNA expression levels of caspase-3, cytochrome C, Bax and Bcl-2 in H9c2 were calculated relatively according to that of GAPDH with the 2^−△△Ct^ method. All the primers for target genes studied and housekeeping genes were listed as following:

GAPDH (120 bp), forward: 5′-AGCCCAGAACATCATCCCTG-3′,

reverse: 5′-ACGGATACATTGGGGGTAGG-3′;

caspase-3 (178 bp), forward: 5′-TCCTGGAGAAATTCAAGGGA-3′,

reverse: 5′-ACGGGATCTGTTTCTTTGCA-3′;

cytochrome C (140 bp), forward: 5′-CCTTTGTGGTGTTGACCAGC-3′,

reverse: 5′-CCATGGAGGTTTGGTCCAGT-3′.

Bax (154 bp), forward: 5′-ATGTGGATACAGACTCCCCC-3′,

reverse: 5′-ATC AGCTCGGGCACTTTAGT-3′;

Bcl-2 (264 bp), forward: 5′-AGGGGCTACGAGTGGGATAC-3′,

reverse: 5′-GGACATCTCTGCAAAGTCGC-3′;

### Statistical analysis

All statistical analyses were performed using Statistical Product and Service Solutions (SPSS) 19.0 software package for Windows (IBM, USA). Values were presented as the mean ± standard deviation (SD). Statistical significance was performed by one-way analysis of variance (ANOVA). Values with *p* < 0.05 were considered to be statistically significant.

## Results

### UPLC–ESI–QTOF MS/MS analysis of the main components of SCBPE

**Fig. 1:**
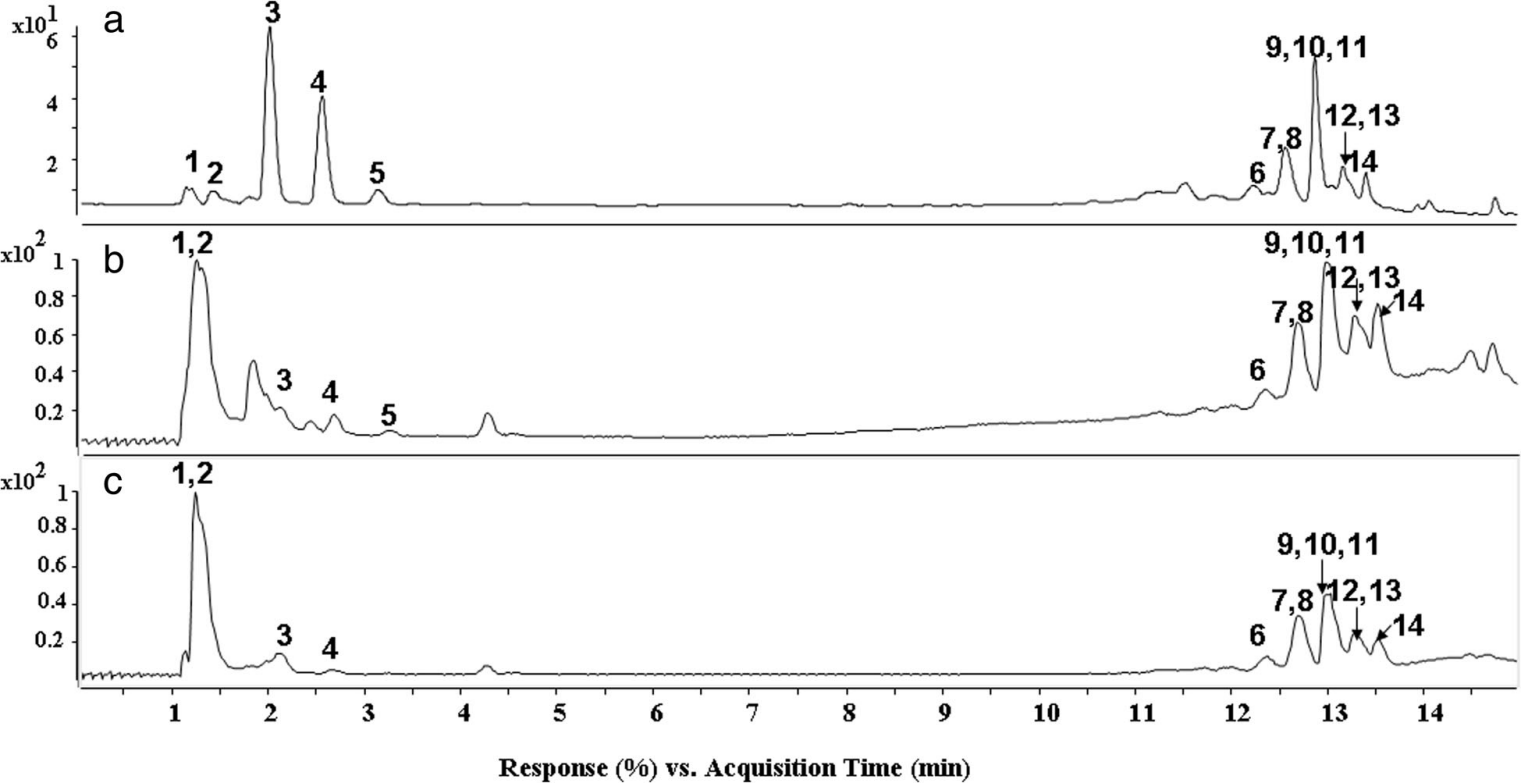
UV chromatogram at 254 nm (**a**), TIC chromatograms in positive ion mode (**b**) and in negative ion mode (**c**) of SCBPE

The UV chromatogram at 254 nm and total ion current (TIC) chromatograms in positive and negative ion modes of SCBPE are shown in Fig. 1. The data on retention time (*t*_R_), [M + H]^+^ (or [M + Na]^+^) ions, [M − H]^−^ ions, molecular weight, double bond equivalences (DBE), and formula are summarized in Table 1. Q–TOF MS/MS data in negative ESI mode of the components detected in SCBPE are also summarized. Fourteen main components, including two carbohydrates, three nucleosides, and nine quinic acid nitrogen-containing derivatives, were identified or tentatively characterized. Peaks 2, 3, 4, and 5 were confirmed to be sucrose, uridine, adenosine, and guanosine, respectively, by comparing their *t*_R_, MS, and MS/MS data with those of the four reference compounds.

**Table 1:** Peak assignments for the analysis of SCBPE

Peak No.	***t***_**R**_ (min)	Identification	(+) ESI-MS ***m/z***		(−) ESI-MS ***m/z***		DBE	Formula
			Observed	Calculated (Δppm)	Observed	Calculated (Δppm)		
1	1.239	Gluconic acid			195.05159	195.05103 (−2.89)	1	C_6_H_12_O_7_
2	1.355	Sucrose ^a^	[M + Na]^+^ 365.10556	365.10543 (0.36)	341.11044	341.10894 (−4.41)	2	C_12_H_22_O_11_
3	2.003	Uridine ^a^	[M + Na]^+^ 267.05951	267.05876 (2.81)	243.06264	243.06226 (−1.56)	5	C_9_H_12_N_2_O_6_
4	2.570	Adenosine ^a^	268.10476	268.10403 (2.71)	266.08973	266.08948 (−0.94)	7	C_10_H_13_N_5_O_4_
5	3.130	Guanosine ^a^	284.10797	284.11018 (−7.78)	282.08436	282.08439 (0.11)	7	C_10_H_13_N_5_O_5_
6	12.366	Caffeoyl-feruloylquinic acid or caffeoyl-isoferuloylquinic acid nitrogen-containing derivative	706.29816	706.29704 (1.60)	704.28380	704.28249 (1.86)	18	C_37_H_43_N_3_O_11_
7	12.714	Caffeoyl-feruloylquinic acid or caffeoyl-isoferuloylquinic acid nitrogen-containing derivative	706.29697	706.29704 (−0.09)	704.28298	704.28249 (0.70)	18	C_37_H_43_N_3_O_11_
8	12.797	Caffeoyl-3′-methoxycinnamylquinic acid or caffeoyl-4′-methoxycinnamylquinic acid nitrogen-containing derivative	690.30371	690.30212 (2.30)	688.28736	688.28757 (−0.31)	18	C_37_H_43_N_3_O_10_
9	12.979	Caffeoyl-feruloylquinic acid or caffeoyl-isoferuloylquinic acid nitrogen-containing derivative	706.29629	706.29704 (−1.06)	704.28123	704.28249 (−1.79)	18	C_37_H_43_N_3_O_11_
10	13.079	Caffeoyl-3′-methoxycinnamylquinic acid or caffeoyl-4′-methoxycinnamylquinic acid nitrogen-containing derivative	690.29517	690.30212 (−10.07)	688.28813	688.28757 (0.81)	18	C_37_H_43_N_3_O_10_
11	13.178	*p* (or *m*)-Coumaroyl-3′-methoxycinnamylquinic acid or *p* (or *m*)-coumaroyl-4′-methoxycinnamylquinic acid nitrogen-containing derivative	674.30984	674.30721 (3.90)	672.29420	672.29266 (2.29)	18	C_37_H_43_N_3_O_9_
12	13.277	Caffeoyl-3′-methoxycinnamylquinic acid or caffeoyl-4′-methoxycinnamylquinic acid nitrogen-containing derivative	690.30160	690.30212 (−0.75)	688.28800	688.28757 (0.63)	18	C_37_H_43_N_3_O_10_
13	13.244	*p* (or *m*)-Coumaroyl-3′-methoxycinnamylquinic acid or *p* (or *m*)-coumaroyl-4′-methoxycinnamylquinic acid nitrogen-containing derivative	674.30817	674.30721 (1.42)	672.29314	672.29266 (0.71)	18	C_37_H_43_N_3_O_9_
14	13.526	*p* (or *m*)-Coumaroyl-3′-methoxycinnamylquinic acid or *p* (or *m*)-coumaroyl-4′-methoxycinnamylquinic acid nitrogen-containing derivative	674.30941	674.30721 (3.26)	672.29376	672.29266 (1.64)	18	C_37_H_43_N_3_O_9_

^a^compared with standard compounds

Peak 1 gave an [M − H]^−^ ion at *m/z* 195.05159, suggesting that the formula was C_6_H_12_O_7_ with DBE of 1. It yielded the [M − H − H_2_O]^−^ ion at *m/z* 177.03972, [M − H − 2H_2_O]^−^ ion at *m/z* 159.02981, and [M − H − 2H_2_O − CH_2_O]^−^ ion at *m/z* 129.01928 (Additional file 1), which were also found in the MS/MS spectrum of gluconic acid in a previous study [28]. Therefore, according to the formula, DBE, and MS/MS data, peak 1 was tentatively identified as gluconic acid.

**Fig. 2:**
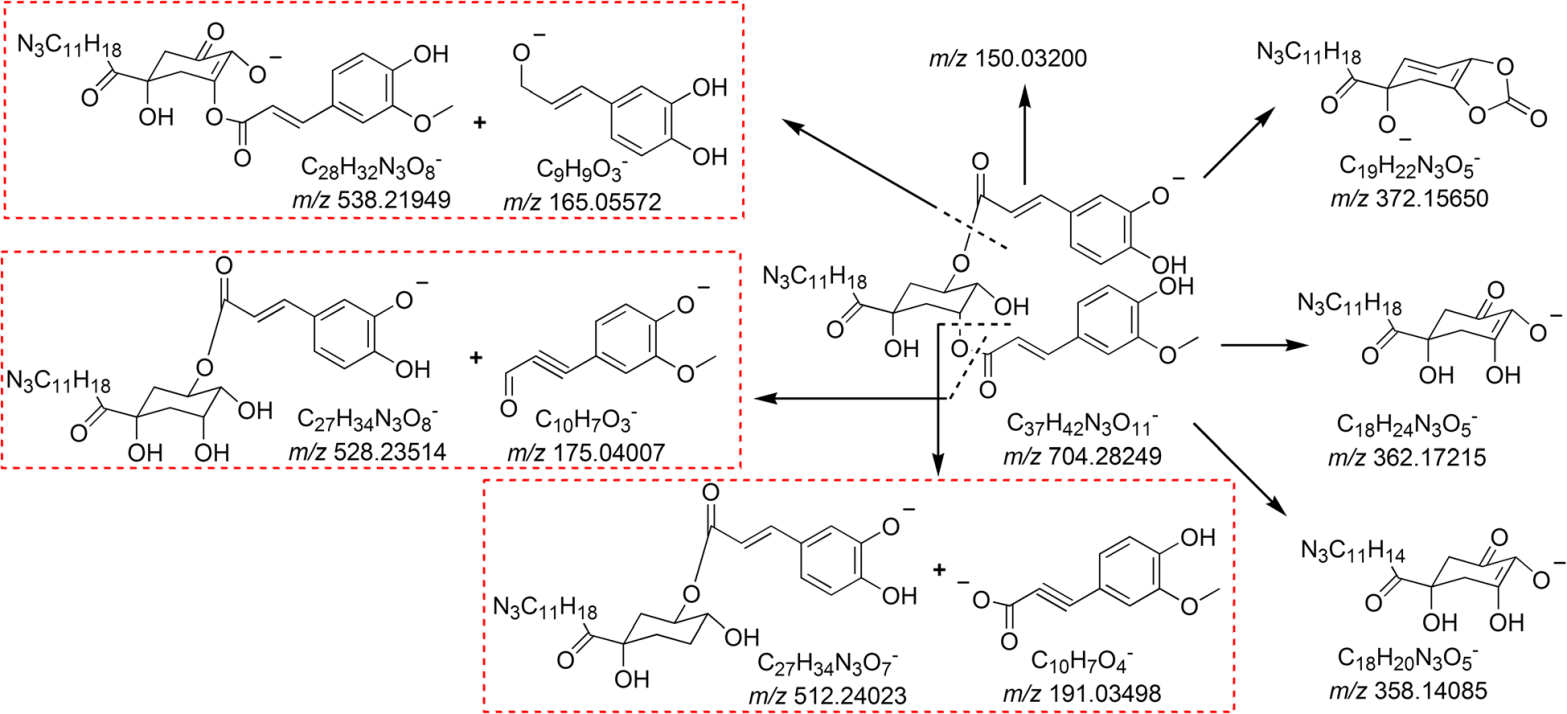
The proposed (−)ESI-Q-TOF MS/MS fragmentation pathway of 3-caffeoyl-5-feruloylquinic acid nitrogen-containing derivative

Peaks 6, 7, and 9 generated [M − H]^−^ ions at *m/z* 704.28380, 704.28298, and 704.28123, respectively, as well as [M + H]^+^ ions at *m/z* 706.29816, 706.29697, and 706.29629, respectively, indicating that they were isomers with the formula of C_37_H_43_N_3_O_11_. In addition, their MS/MS spectra were identical, suggesting that the three components could possess the same nuclear structure and substituent groups, but the substituted sites of their substituent groups could be different. The (−)ESI–Q–TOF MS/MS spectrum of peak 9 is summarized in Additional file 1 and shown in Additional file 2A. The prominent ions at *m/z* 538 (base peak) and *m/z* 165 were a pair of product ions. The formula of the *m/z* 165 ion was C_9_H_9_O_3_^−^, indicating that a caffeoyl group might exist in the structure. Moreover, the product ions at *m/z* 175 and *m/z* 191 corresponded to C_10_H_7_O_3_^−^ and C_10_H_7_O_4_^−^ ions, respectively, suggesting that another substituent group could be a feruloyl group or an isoferuloyl group; similarly, the ions at *m/z* 528 and *m/z* 512 were their corresponding ions, respectively. The *m/z* 362 product ion with the formula of C_18_H_24_N_3_O_5_^−^ suggested that a quinic acid nuclear structure was substituted with a C_11_H_18_N_3_ group. However, numerous substituted sites were found on quinic acid; thus, determining the specific substituted sites according to the MS/MS data was difficult. Hence, peaks 6, 7, and 9 were preliminarily characterized as caffeoyl–feruloylquinic acid or caffeoyl–isoferuloylquinic acid nitrogen-containing derivatives. Nevertheless, these characterization results warrant further investigation. The proposed fragmentation pathway of 3-caffeoyl-5-feruloylquinic acid nitrogen-containing derivative in the negative ion mode is shown in Fig. 2.

**Fig. 3:**
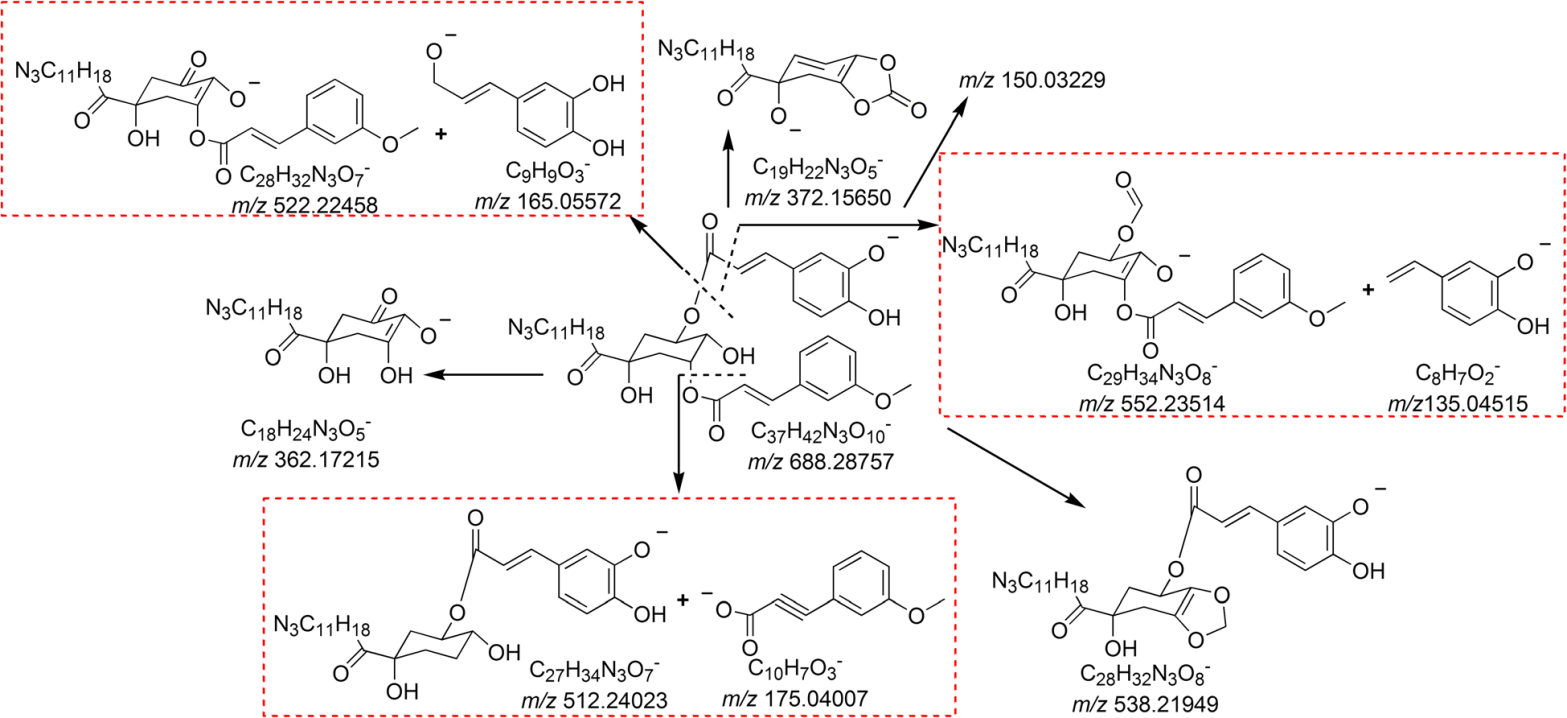
The proposed (−)ESI-Q-TOF MS/MS fragmentation pathway of 3-caffeoyl-5-3′-methoxycinnamylquinic acid nitrogen-containing derivative

Peaks 8, 10, and 12 gave [M − H]^−^ ions at *m/z* 688.28736, 688.28813, and 688.28800, respectively, as well as [M + H]^+^ ions at *m/z* 690.30371, 690.29517, and 690.30160, respectively, suggesting that the formula of the three isomers was C_37_H_43_N_3_O_10_ with a molecular weight 16 Da lower than that of peaks 6, 7, and 9. Moreover, these peaks could be classified as quinic acid nitrogen-containing derivatives. Furthermore, the three components had identical MS/MS spectra. The (−)ESI–Q–TOF MS/MS spectrum of peak 12 is summarized in Additional file 1 and shown in Additional file 2B. The ions at *m/z* 165 and *m/z* 522 were a pair of product ions. The formula of the *m/z* 165 ion was C_9_H_9_O_3_^−^, indicating that a caffeoyl group might exist in the structure. The product ion at *m/z* 175 was attributed to C_10_H_7_O_3_^−^ ion, suggesting that the other substituent group could be a 3′-methoxycinnamyl group or a 4′-methoxycinnamyl group, and the ion at *m/z* 512 was its corresponding ion. Thus, peaks 8, 10, and 12 could potentially be caffeoyl-3′-methoxycinnamylquinic acid or caffeoyl-4′-methoxycinnamylquinic acid nitrogen-containing derivatives. The proposed fragmentation pathway of 3-caffeoyl-5-3′-methoxycinnamylquinic acid nitrogen-containing derivative in the negative ion mode is shown in Fig. 3.

Peaks 11, 13, and 14 generated deprotonated molecule [M − H]^−^ ions at *m/z* 672.29420, 672.29314, and 672.29376, respectively, as well as [M + H]^+^ ions at *m/z* 674.30984, 674.30817, and 674.30941, respectively, indicating that the formula of the three isomers was C_37_H_43_N_3_O_9_ with a molecular weight 16 Da lower than that of peaks 8, 10, and 12. Moreover, they might belong to quinic acid nitrogen-containing derivatives. In addition, the three components had identical MS/MS spectra. The (−)ESI–Q–TOF MS/MS spectrum of peak 14 is summarized in Additional file 1 and shown in Additional file 2C. The *m/z* 522 ion corresponding to the base peak and the *m/z* 149 ion were a pair of product ions. The formula of the *m/z* 149 ion was C_9_H_9_O_2_^−^, indicating that a *p*-coumaroyl or *m*-coumaroyl group might exist in the structure. The product ion at *m/z* 175 was the same as that of peaks 8, 10, and 12 in the MS/MS spectra, suggesting that the other substituent group could be a 3′-methoxycinnamyl group or a 4′-methoxycinnamyl group, and the ion at *m/z* 496 was its corresponding ion. Thus, peaks 11, 13, and 14 were tentatively assigned as *p* (or *m*)-coumaroyl-3′-methoxycinnamylquinic acid or *p* (or *m*)-coumaroyl-4′-methoxycinnamylquinic acid nitrogen-containing derivatives. The proposed fragmentation pathway of 3–3′-methoxycinnamyl-5-*p*-coumaroylquinic acid nitrogen-containing derivative in the negative ion mode is shown in Additional file 3.

To the best of our knowledge, the present study is the first to identify these nine quinic acid nitrogen-containing derivatives from bee pollen.

### Quantitative analysis of the three nucleosides of SCBPE via UPLC–DAD

The baseline resolution of uridine, guanosine, and adenosine was obtained (Additional file 4). The calibration curves were *y* = 12.114*x* + 7.4817 for uridine, *y* = 12.916*x* + 6.6416 for guanosine, and *y* = 10.811*x* + 4.1441 for adenosine, and they all displayed good linearity (*R*^2^ ≥ 0.9999). The LOD of the three nucleosides was 0.195 μg/mL. The LOQ of uridine and guanosine was 1.56 μg/mL, whereas that of adenosine was 0.78 μg/mL. The RSD values of precision were 0.57% for uridine, 0.91% for guanosine, and 0.92% for adenosine, whereas those of repeatability were 1.78% for uridine, 6.93% for guanosine, and 2.14% for adenosine. These values indicated that the method had good precision and repeatability. The RSD values of stability were 1.51% for uridine, 6.85% for guanosine, and 1.96% for adenosine, suggesting that the sample had good stability within 48 h. The average recovery for uridine was 92.87% with RSD of 2.27%, that for guanosine was 99.68% with RSD of 4.99%, and that for adenosine was 102.63% with RSD of 6.31%, demonstrating that the method developed herein was reproducible with good accuracy. The three nucleosides from six SCBPE samples were quantified using this validated method. The contents of uridine, guanosine, and adenosine were 2.4945 ± 0.0185, 0.1896 ± 0.0049, and 1.8418 ± 0.0157 μg/mg, respectively.

### Screening of the appropriate concentration and administration time of SCBPE in vitro

The effects of SCBPE on H9c2 cardiomyocytes for 72 h without H_2_O_2_ treatment were studied. The OD values (*n* = 5) of the negative control group and 6.25, 12.5, 25, 50, 100, 250, and 500 μg/mL SCBPE groups were 0.6175 ± 0.0034, 0.5692 ± 0.0031, 0.5203 ± 0.0079, 0.5524 ± 0.0078, 0.5437 ± 0.0056, 0.5072 ± 0.0102, 0.5404 ± 0.0012, and 0.5533 ± 0.0011, respectively. No significant differences were observed among these groups, suggesting that these SCBPE concentrations could not affect the viability of H9c2 cardiomyocytes. The appropriate concentration and administration time of SCBPE that have an effect on cell vitality were screened via MTT assay. Seven SCBPE concentrations, namely, 6.25, 12.5, 25, 50, 100, 250, and 500 μg/mL, and three time periods of 24, 48, and 72 h were tested. As shown in Additional file 5, the OD values of the H_2_O_2_ group significantly decreased compared with those of the negative control group (*p* < 0.01), suggesting that oxidative injury in H9c2 cells had been induced by 400 μM H_2_O_2_ for 2 h. Compared with those of the H_2_O_2_ group, the OD values of the Vc and SCBPE groups pretreated for 48 and 72 h remarkably increased, indicating that Vc and SCBPE could promote cell proliferation. However, the proliferation of myocardial cells pretreated with Vc and SCBPE for 24 h was not evident.

After 48 h pretreatment, both Vc and SCBPE significantly promoted the proliferation of cardiac myocytes (*p* < 0.01). However, the OD values of the Vc and SCBPE groups showed remarkable differences compared with those of the negative control group (*p* < 0.01). By contrast, the proliferation of H_2_O_2_-damaged myocardial cells pretreated with Vc and SCBPE (except 500 μg/mL) for 72 h was significantly promoted (*p* < 0.01), and the OD values of SCBPE groups (except 250 and 500 μg/mL) did not show significant differences compared with those of the negative control group. On the basis of these results, the drug administration for 72 h and concentrations of 12.5, 25, and 50 μg/mL SCBPE were selected for subsequent experiments.

### Effects of SCBPE on the survival rate and morphology of H_2_O_2_-induced H9c2 cells

As shown in Additional file 6**,** the survival rate of myocardial cells in the H_2_O_2_ group was significantly lower than that in the negative control groups (*p* < 0.01). Compared with that of the H_2_O_2_ group, the cell viability of the Vc group and all the three SCBPE groups significantly increased (*p* < 0.01). Meanwhile, the cell survival rate for 12.5 and 50 μg/mL SCBPE groups was approximate to that for the Vc groups. However, the survival rate for the 25 μg/mL SCBPE group was significantly higher than that for the Vc group (*p* < 0.05).

**Fig. 4:**
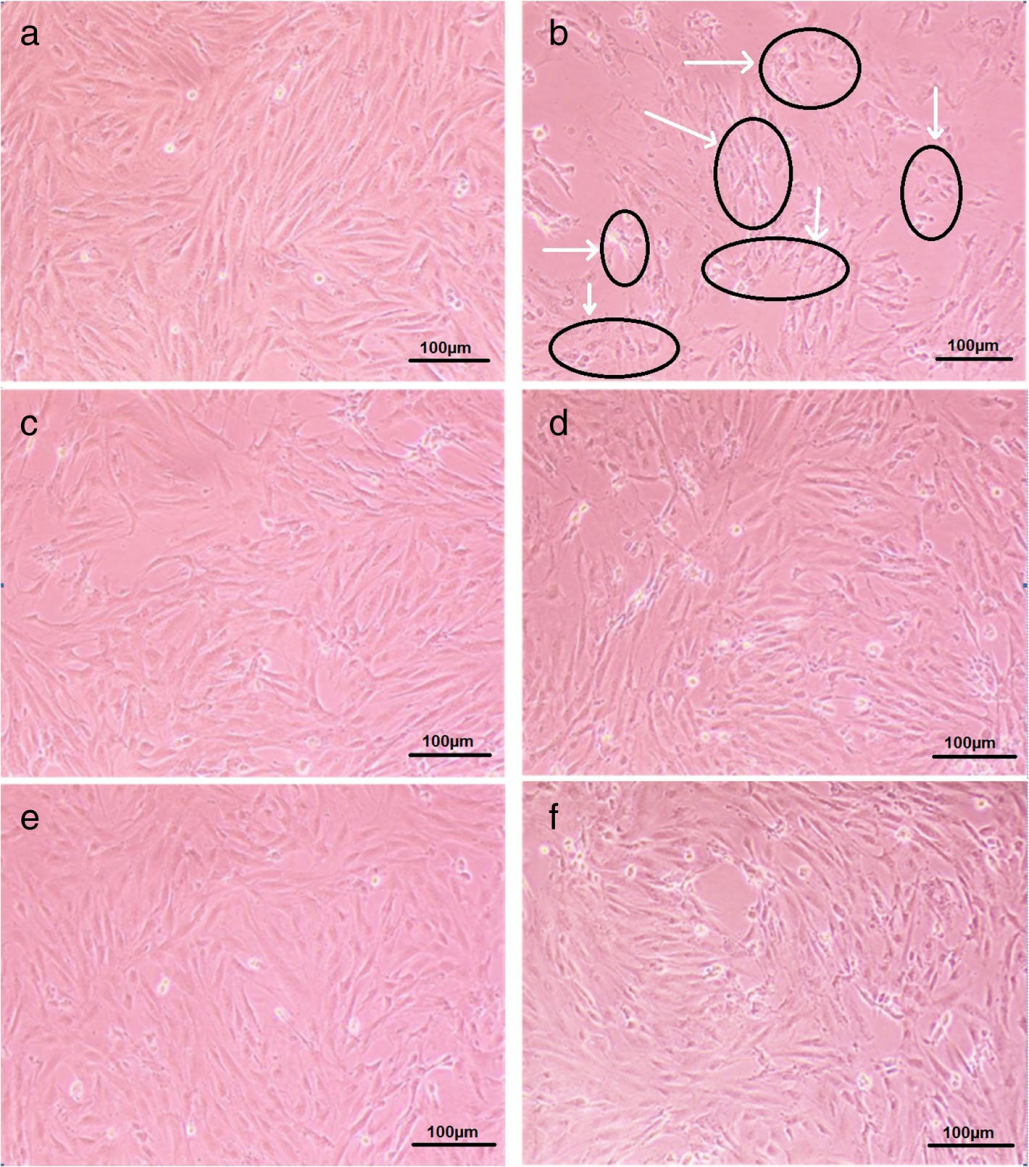
The effects of SCBPE on morphology of H9c2 myocardial cells. **a**, negative control group; **b**, H_2_O_2_ group; **c**, positive control (Vc) group; **d**, 12.5 μg/mL SCBPE; **e**, 25 μg/mL SCBPE; **f**, 50 μg/mL SCBPE

Microscopic observation revealed that the cell morphology of the negative control group was good, and no abnormal changes were observed. By contrast, microscopic observation showed partial shrinkage of myocardial cells in the H_2_O_2_ group. Moreover, the nucleus and cytoplasm vacuoles were dim, the pseudopod disappeared, and intercellular spaces significantly increased. In the Vc and SCBPE groups, Vc and SCBPE had evident reduction effects. The cell morphology and survival rate markedly improved, the shrinking of myocardial cell pseudopodia was not remarkable, and intercellular spaces became smaller and had increased connections (Fig. 4). These results demonstrated the protective effects of SCBPE on H9c2 myocardial cells against H_2_O_2_-induced cell injury.

### Effects of SCBPE on SOD activity and MDA and GSH levels induced by H_2_O_2_ in H9c2 cells

After pretreatment for 72 h by different SCBPE concentrations (12.5, 25, and 50 μg/mL) and 40 μM Vc, H9c2 cells were incubated in 400 μM H_2_O_2_ for 2 h. SOD activity and GSH level were significantly lower (*p* < 0.01) and MDA level was significantly higher (*p* < 0.01) in the H_2_O_2_ group than those in the negative control group. SOD activity and GSH level were significantly higher in the 25 and 50 μg/mL SCBPE groups (*p* < 0.05, *p* < 0.01) than those in the H_2_O_2_ group. Moreover, MDA level was significantly lower in 40 μM Vc and SCBPE groups (*p* < 0.05, *p* < 0.01) than that in the H_2_O_2_ group (Table 2).

**Table 2:** Effects of SCBPE on SOD activity and MDA and GSH levels induced by H_2_O_2_ in H9c2 cells

Group	SOD (U/mL)^a^	GSH (μmol/g prot)^a^	MDA (nmol/mg prot)^a^
Negative control group	54.6014 ± 0.2399*	39.8462 ± 1.6048*	0.3034 ± 0.1045*
H_2_O_2_ group	51.2855 ± 0.4685	26.3654 ± 5.7993	1.4215 ± 0.3413
Positive control (Vc) group	51.9344 ± 0.7776	26.1652 ± 1.3582	0.5677 ± 0.1884*
12.5 μg/mL SCBPE	52.1944 ± 0.5090	36.4072 ± 0.4569#	0.6326 ± 0.1262#
25 μg/mL SCBPE	53.3966 ± 0.4482#	41.3148 ± 2.3994*	0.4958 ± 0.0945*
50 μg/mL SCBPE	52.9136 ± 0.3818#	35.6945 ± 2.7386#	0.5665 ± 0.1061*

^a^Values represent mean ± SD of three independent experiments, and *n* = 6 in each experiment. Compared with H_2_O_2_ group, #*p *< 0.05, *p < 0.01

### Effects of SCBPE on apoptosis of H9c2 myocardial cells induced by H_2_O_2_

**Fig. 5:**
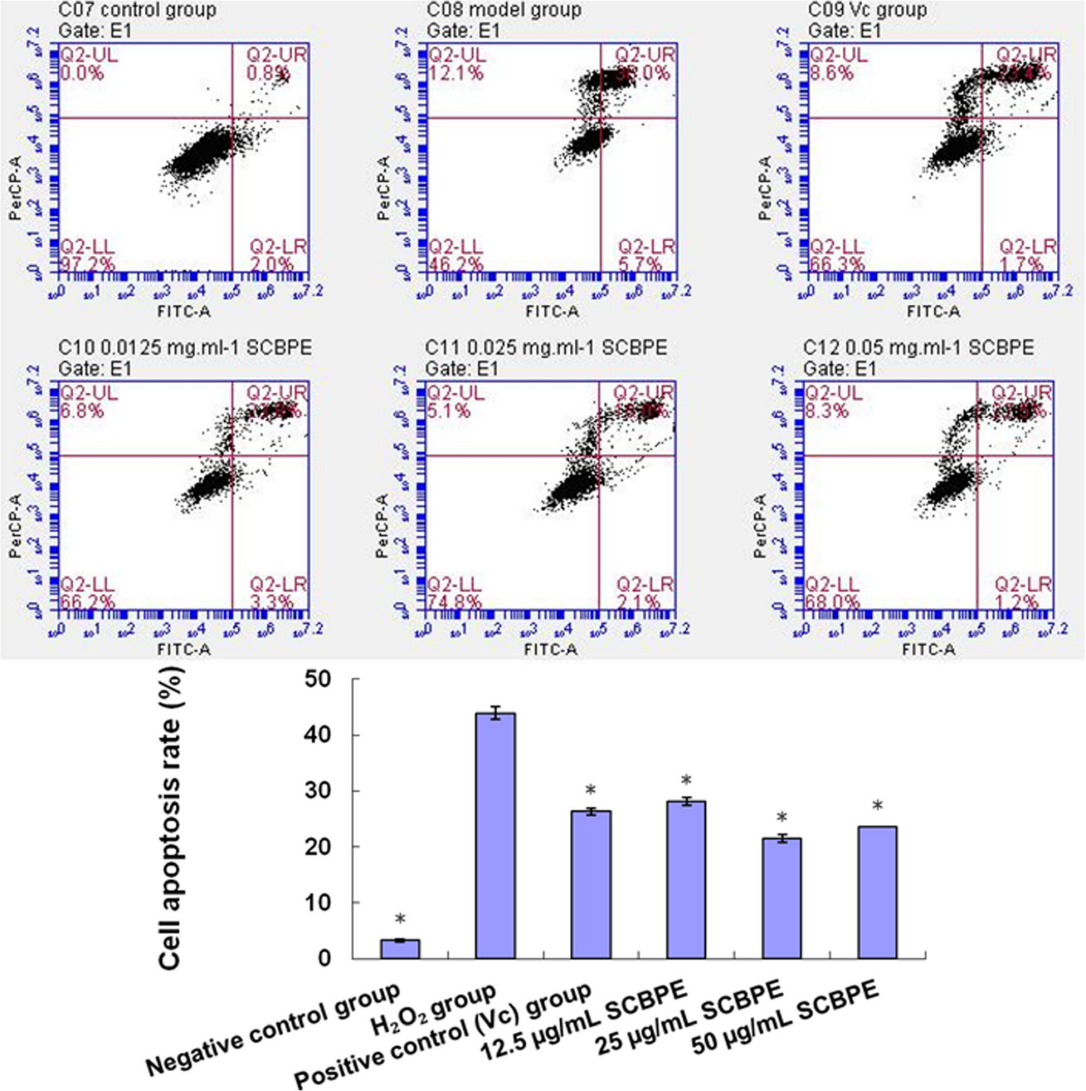
Effect of SCBPE on apoptosis of H9c2 cardiomyocytes induced by H_2_O_2_. C07, negative control group; C08, H_2_O_2_ group; C09, positive control (Vc) group; C10, 12.5 μg/mL SCBPE; C11, 25 μg/mL SCBPE; C12, 50 μg/mL SCBPE. * Compared with H_2_O_2_ group, *p* < 0.01

The antiapoptotic effects of SCBPE on H9c2 cardiomyocytes against H_2_O_2_-induced injury as quantified by the percentage of apoptotic cells were detected by Annexin V-FITC/PI double staining via flow cytometry. As shown in Fig. 5, after 2 h of 400 μM H_2_O_2_ damage, the apoptosis rate significantly increased in the H_2_O_2_ group (43.93%) compared with that in the negative control group (3.3%) (*p* < 0.01), whereas that of H9c2 cardiomyocytes preincubated with 40 μM Vc and 12.5, 25, and 50 μg/mL SCBPE for 72 h prior to H_2_O_2_ exposure significantly decreased compared with that in the H_2_O_2_ group (*p* < 0.01); the percentage of apoptotic cells was 26.33, 28.17, 21.53, and 23.6%, respectively. These results demonstrated that SCBPE inhibited H_2_O_2_-induced apoptosis in H9c2 cardiomyocytes.

### Effects of SCBPE on the mRNA expressions of caspase-3, cytochrome C, Bax, and Bcl-2 of H9c2 myocardial cells induced by H_2_O_2_

**Fig. 6:**
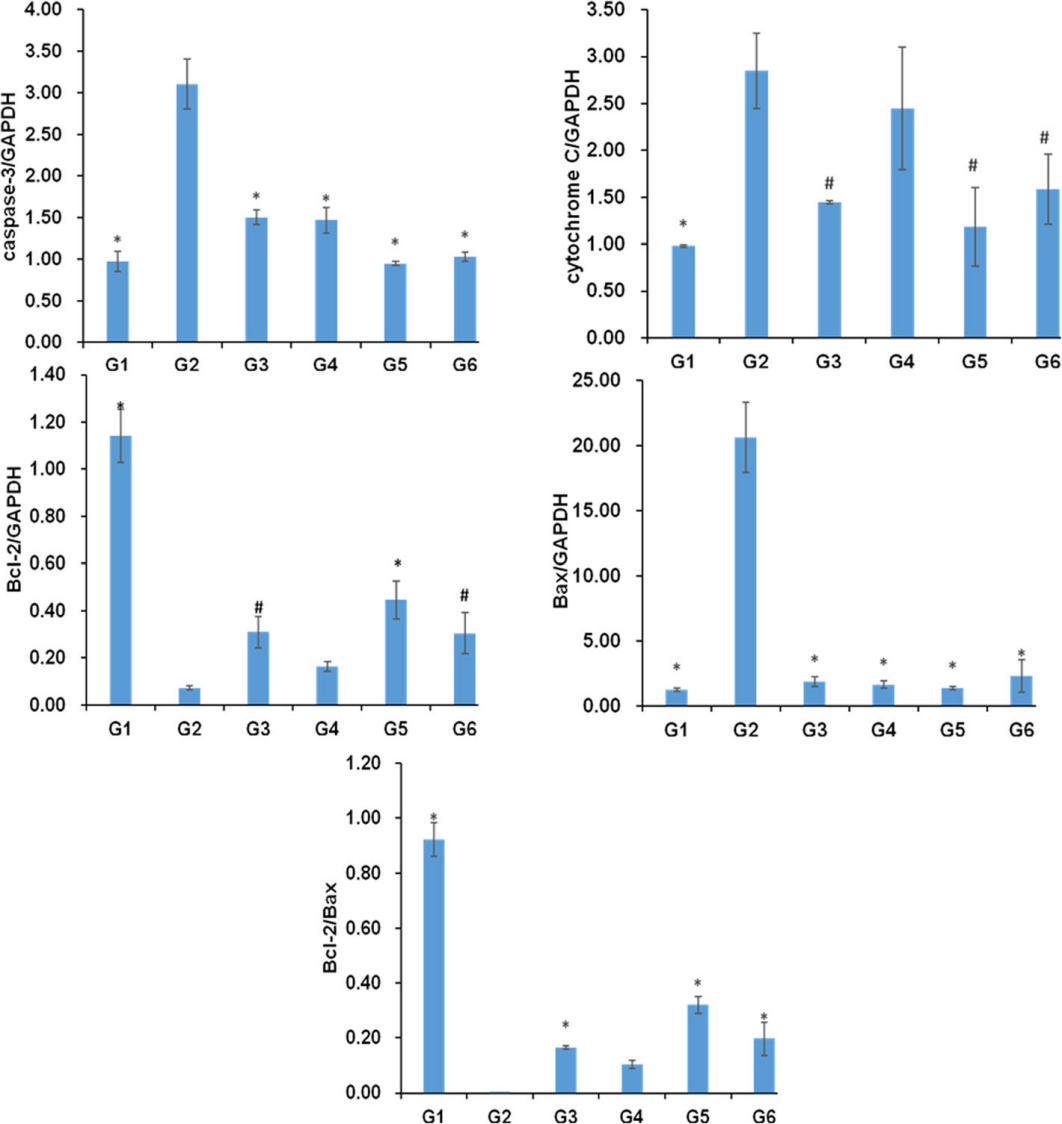
The effect of SCBPE on the mRNA expression of caspase-3, cytochrome C, Bcl-2, Bax, and Bcl-2/Bax induced by H_2_O_2_ in H9c2 myocardial cells of each group. G1, negative control group; G2, H_2_O_2_ group; G3, positive control (Vc) group; G4, 12.5 μg/mL SCBPE; G5, 25 μg/mL SCBPE; G6, 50 μg/mL SCBPE. Compared with H_2_O_2_ group, # *p* < 0.05, * *p* < 0.01

The effects of SCBPE on the mRNA expressions of caspase-3, cytochrome C, Bax, and Bcl-2 on H_2_O_2_-injured H9c2 cells are illustrated in Fig. 6. The mRNA levels of caspase-3, cytochrome C, and Bax were significantly upregulated and that of Bcl-2 was downregulated in the H_2_O_2_ group compared with those in the negative controlgroup (*p* < 0.01). Furthermore, the mRNA level ratio of Bcl-2 to Bax markedly decreased. Compared with those in the H_2_O_2_ group, the mRNA levels of caspase-3, cytochrome C, and Bax were significantly downregulated and that of Bcl-2 was upregulated in the SCBPE groups. Moreover, the mRNA level ratio of Bcl-2 to Bax significantly increased. These results suggested that SCBPE could play a protective role against H_2_O_2_-induced H9c2 cell injury by regulating the mRNA expressions of caspase-3, cytochrome C, Bax, and Bcl-2, thereby reducing the cell apoptosis caused by H_2_O_2_.

## Discussion

On the basis of the results of cell survival rate measurements and morphological observations, the present study demonstrated the protective effects of SCBPE on H9c2 cardiomyocytes against H_2_O_2_-induced cell injury. SOD activity and GSH level are also important lines of defense against free radicals [29, 30]. MDA, one of the end products of lipid peroxidation, could lead to severe cell damage by causing polymerization and crosslinking of membrane components, which are recognized as an indirect oxidative stress marker of cellular injury [11, 31]. Accordingly, SOD activity and GSH and MDA levels are usually used in assessing oxidative stress [27, 30]. In this study, GSH content and SOD activity significantly increased and MDA content decreased in H9c2 cardiomyocytes in the SCBPE groups compared with those in the H_2_O_2_ group, suggesting that the protective effects of SCBPE against H_2_O_2_-induced myocardial cell injury could be related to antioxidant activity. These results were consistent with those of our previous research in which we reported that SCBPE could exert antioxidative and cardioprotective effects on isoprenaline-induced myocardial infarction in rats [18].

In addition, two carbohydrates, three nucleosides, and nine quinic acid nitrogen-containing derivatives were identified or tentatively characterized via UPLC–QTOF MS/MS in the present study. The three nucleosides were quantitatively analyzed via UPLC–DAD to reveal the main components of SCBPE. Among the three nucleosides, the contents of adenosine and uridine in SCBPE were 1.8418 ± 0.0157 and 2.4945 ± 0.0185 μg/mg, respectively, which were about 10 and 13 times larger than that of guanosine with 0.1896 ± 0.0049 μg/mg, respectively. Administrating an intracoronary infusion of adenosine at a rate of 3.75 mg/min for the first hour of reperfusion after a 90 min left anterior descending occlusion remarkably reduces infarct size and improves regional ventricular function in the ischemic zone in canine preparation [32]. Moreover, intravenous infusion of 0.15 mg/kg/min adenosine results in a sustained reduction in infarct size in a canine model [33]. The Acute Myocardial Infarction Study of Adenosine (AMISTAD) trial by intravenous infusion of adenosine at 70 mg/kg/min for 3 h and AMISTAD-II trial by intravenous infusion of adenosine at 50 mg/kg/min or 70 mg/kg/min for 3 h both demonstrated that adenosine administration with reperfusion therapy can reduce infarct size and improve ventricular function [34–36]. Intravenous infusion of 30 mg/kg uridine can reduce the incidence of ventricular tachycardia and ventricular fibrillation, whereas infusion of uridine during the entire period of acute myocardial infarction (60 min) would probably contribute to manifestation of antioxidant and anti-ischemic actions [37]. In the present study, nine quinic acid nitrogen-containing derivatives were identified from bee pollen for the first time. Except for the nitrogen-containing group with the molecular composition of C_11_N_3_H_18_ in the structure, the other substituent groups on quinic acid, namely, caffeoyl, feruloyl (or isoferuloyl), 3′-methoxycinnamyl (or 4′-methoxycinnamyl), and *p*-coumaroyl (or *m*-coumaroyl) groups, have multiple unsaturated groups in their structure. The presence of these unsaturated groups possibly results in favorable antioxidative capacity. For instance, the caffeoyl–quinic acid group in the structures of peaks 6–10 and 12 reportedly has antioxidative activities [38] and exerts protective effect on cardiomyocytes against oxidative stress-induced damage [10]. Furthermore, the protective effects of uridine, guanosine, adenosine, and quinic acid nitrogen-containing derivatives on H9c2 myocardial cells against H_2_O_2_-induced cell injury were studied. The cell viability determined via MTT assay is shown in Additional file 7. Compared with those in the H_2_O_2_ group, the OD values significantly increased in the adenosine groups (0.2–100 μg/mL for 48 h and 0.78–25 [except for 6.25 μg/mL] for 72 h), uridine groups (100 μg/mL for 48 h and 25–100 μg/mL for 72 h), guanosine groups (0.2–3.125 μg/mL for 48 h and 0.39 and 1.56 μg/mL for 72 h), and nitrogen-containing quinic acid derivative groups (0.2–12.5 μg/mL for 48 h and 0.2–6.25 μg/mL for 72 h). Therefore, the main nucleosides and quinic acid nitrogen-containing derivatives could be the main active components of SCBPE against H_2_O_2_-induced H9c2 cardiac cell injury.

Oxidative stress mediated by ROS can trigger myocyte apoptosis, which could be one of the major mechanisms in myocardial ischemia injury [9, 39]. Once ROS are generated, they can increase mitochondrial permeability, resulting in cytochrome C release. Bcl-2 family member proteins can regulate the release of mitochondrial cytochrome C during oxidative stress in cardiomyocytes, and cell survival may increase when Bcl-2 expression is relatively high but only when Bax expression is low [40]. Hence, the ratio of Bax to Bcl-2 is an effective predictor of the apoptotic fate of cardiomyocytes [41, 42]. Subsequently, cytochrome C can form a complex with caspase-9 and APAF-1, leading to caspase-9 activation. In turn, caspase-9 can activate the executioner caspases (caspase-3, − 6, and − 7) and induce cell death [10, 43]. Several studies reported that H_2_O_2_ exposure can induce cardiomyocyte apoptosis [39] and substantially increase the levels of Bax and caspase-3 and decrease the level of Bcl-2 in H9c2 cells [40]. The present study demonstrated that SCBPE remarkably inhibited cardiomyocyte apoptosis induced by H_2_O_2_ by downregulating Bax, cytochrome C, and caspase-3 mRNA expression and upregulating Bcl-2 mRNA expression.

## Conclusion

In this study, two carbohydrates, three nucleosides, and nine quinic acid nitrogen-containing derivatives were identified or tentatively characterized in SCBPE via UPLC–QTOF MS/MS. The nine quinic acid nitrogen-containing derivatives were reported in bee pollen for the first time. Three nucleosides, namely, uridine, guanosine, and adenosine, were quantitatively analyzed via UPLC–DAD. Results showed that SCBPE could reduce oxidative stress-induced apoptosis in H9c2 cells by downregulating Bax, cytochrome C, and caspase-3 mRNA expression and upregulating Bcl-2 mRNA expression. These findings indicated that SCBPE exerts protective effects on H9c2 cells against oxidative stress and apoptosis in vitro, and the main nucleosides and quinic acid nitrogen-containing derivatives could be the primary pharmacodynamic substances involved in this mechanism.

## Supplementary information

**Additional file 1:.** (−)ESI-Q-TOF MS/MS data of peaks 1, 9, 12 and 14 of SCBPE.

**Additional file 2:.** (−)ESI-Q-TOF MS/MS spectra of peaks 9 (A), 12 (B) and 14 (C).

**Additional file 3:.** The proposed (−)ESI-Q-TOF MS/MS fragmentation pathway of 3–3′-methoxycinnamyl-5-p-coumaroylquinic acid nitrogen-containing derivative.

**Additional file 4:.** UPLC–DAD Chromatograms at 254 nm of the mixed reference substances including 25 μg/mL of uridine, 26.25 μg/mL of guanosine and 26.75 μg/mL of adenosine (A) and SCBPE (B).

**Additional file 5:.** Effect of seven concentrations of SCBPE on proliferation of H9c2 cells induced by H_2_O_2_ in three time periods, respectively.

**Additional file 6: **The effect of SCBPE on cell survival rate of H9c2 cells. * Compared with H_2_O_2_ group, *p* < 0.01. # SCBPE+H_2_O_2_ groups compared with Vc + H_2_O_2_ group, *p* < 0.05.

**Additional file 7:.** Effect of adenosine, guanosine, uridine and nitrogen-containing quininic acid derivatives on H_2_O_2_-induced H9c2 myocardial cell injury, respectively.

## Data Availability

The datasets used or analyzed during the study are available from the corresponding author on reasonable request.

## Abbreviations

DBE: Double bond equivalences
ESI: Electrospray ionization
FITC: Fluorescein isothiocyanate
GSH: Glutathione
H_2_O_2_: Hydrogen peroxide
MDA: Malondialdehyde
MTT: 3-(4,5-dimethylthiazol-2-yl)-2,5-diphenyltetrazolium bromide
OD: Optical density
PI: Propidium iodide
ROS: Reactive oxygen species
RSD: Relative standard deviation
SCBPE: *Schisandra chinensis* bee pollen extract
SOD: Superoxide dismutase
TIC: Total ion current
Vc: Vitamin C
