# Supplementary material for: Schisandra chinensis bee pollen’s chemical profiles and protective effect against H2O2-induced apoptosis in H9c2 cardiomyocytes
Source: BMC Complement Med Ther. 2020 Sep 10;20:274. doi: 10.1186/s12906-020-03069-1 (PMC7487998; doi:10.1186/s12906-020-03069-1)
Supplement: Supplementary file 1 — Additional file 1:. (−)ESI-Q-TOF MS/MS data of peaks 1, 9, 12 and 14 of SCBPE. [file 12906_2020_3069_MOESM1_ESM.doc]

**Additional file 1** (–)ESI-Q-TOF MS/MS data of peaks 1, 9, 12 and 14 of SCBPE.

|  | **(-) ESI-MS^2^ *m/z* (% base peak)** | | | |
| --- | --- | --- | --- | --- |
| Peak | Observed mass | Calculated mass (Δppm) | Proposed formula | DBE |
| 1 | MS^2^[195]: |  |  |  |
|  | 129.01928 (100) | 129.01933 (-0.39) | C_5_H_5_O_4_^-^ | 3.5 |
|  | 159.02981 (3) | 159.02990 (-0.57) | C_6_H_7_O_5_^-^ | 3.5 |
|  | 177.03972 (3) | 177.04046 (-4.18) | C_6_H_9_O_6_^-^ | 2.5 |
|  | 195.05050 (29) | 195.05103 (-2.72) | C_6_H_11_O_7_^-^ | 1.5 |
| 9 | MS^2^[704]: |  |  |  |
|  | 150.03200 (15) | **―**^a^ |  |  |
|  | 165.05574 (56) | 165.05572 (0.12) | C_9_H_9_O_3_^-^ | 5.5 |
|  | 175.03994 (7) | 175.04007 (-0.74) | C_10_H_7_O_3_^-^ | 7.5 |
|  | 191.03506 (6) | 191.03498 (0.42) | C_10_H_7_O_4_^-^ | 7.5 |
|  | 358.14153 (7) | 358.14085 (1.90) | C_18_H_20_N_3_O_5_^-^ | 10.5 |
|  | 362.17290 (25) | 362.17215 (2.07) | C_18_H_24_N_3_O_5_^-^ | 8.5 |
|  | 372.15733 (13) | 372.15650 (2.23) | C_19_H_22_N_3_O_5_^-^ | 10.5 |
|  | 512.24095 (12) | 512.24023 (1.41) | C_27_H_34_N_3_O_7_^-^ | 12.5 |
|  | 528.23576 (9) | 528.23514 (1.17) | C_27_H_34_N_3_O_8_^-^ | 12.5 |
|  | 538.22060 (100) | 538.21949 (2.06) | C_28_H_32_N_3_O_8_^-^ | 14.5 |
|  | 704.28370 (43) | 704.28249 (1.72) | C_37_H_42_N_3_O_11_^-^ | 18.5 |
| 12 | MS^2^[688]: |  |  |  |
|  | 135.0442 (6) | 135.04515 (-7.03) | C_8_H_7_O_2_^-^ | 5.5 |
|  | 150.03229 (12) | **―**^a^ |  |  |
|  | 165.05537 (49) | 165.05572 (-2.12) | C_9_H_9_O_3_^−^ | 5.5 |
|  | 175.03986 (9) | 175.04007 (-1.20) | C_10_H_7_O_3_^-^ | 7.5 |
|  | 362.17242 (7) | 362.17215 (0.75) | C_18_H_24_N_3_O_5_^−^ | 8.5 |
|  | 372.15555 (17) | 372.15650 (-2.55) | C_19_H_22_N_3_O_5_^−^ | 10.5 |
|  | 512.24049 (28) | 512.24023 (0.51) | C_27_H_34_N_3_O_7_^−^ | 12.5 |
|  | 522.22423 (78) | 522.22458 (-0.67) | C_28_H_32_N_3_O_7_^−^ | 14.5 |
|  | 538.21847 (21) | 538.21949 (-1.90) | C_28_H_32_N_3_O_8_^−^ | 14.5 |
|  | 552.23648 (6) | 552.23514 (2.43) | C_29_H_34_N_3_O_8_^-^ | 14.5 |
|  | 688.28651 (100) | 688.28757 (-1.54) | C_37_H_42_N_3_O_10_^−^ | 18.5 |
| 14 | MS^2^[672]: |  |  |  |
|  | 134.03780 (10) | **―**^a^ |  |  |
|  | 135.04506 (15) | 135.04515 (-0.67) | C_8_H_7_O_2_^-^ | 5.5 |
|  | 149.06085 (29) | 149.06080 (0.34) | C_9_H_9_O_2_^-^ | 5.5 |
|  | 175.04073 (14) | 175.04007 (3.77) | C_10_H_7_O_3_^-^ | 7.5 |
|  | 372.15707 (23) | 372.15650 (1.53) | C_19_H_22_N_3_O_5_^−^ | 10.5 |
|  | 496.24616 (9) | 496.24531 (1.71) | C_27_H_34_N_3_O_6_^-^ | 12.5 |
|  | 522.22488 (100) | 522.22458 (0.57) | C_28_H_32_N_3_O_7_^−^ | 14.5 |
|  | 536.24057 (10) | 536.24023 (0.63) | C_29_H_34_N_3_O_7_^-^ | 14.5 |
|  | 672.29282 (51) | 672.29266 (0.24) | C_37_H_42_N_3_O_9_^-^ | 18.5 |

^a^ It could not be calculated from the observed mass.
