# Supplementary figures and images for: Schisandra chinensis bee pollen’s chemical profiles and protective effect against H2O2-induced apoptosis in H9c2 cardiomyocytes
Source: BMC Complement Med Ther. 2020 Sep 10;20:274. doi: 10.1186/s12906-020-03069-1 (PMC7487998; doi:10.1186/s12906-020-03069-1)

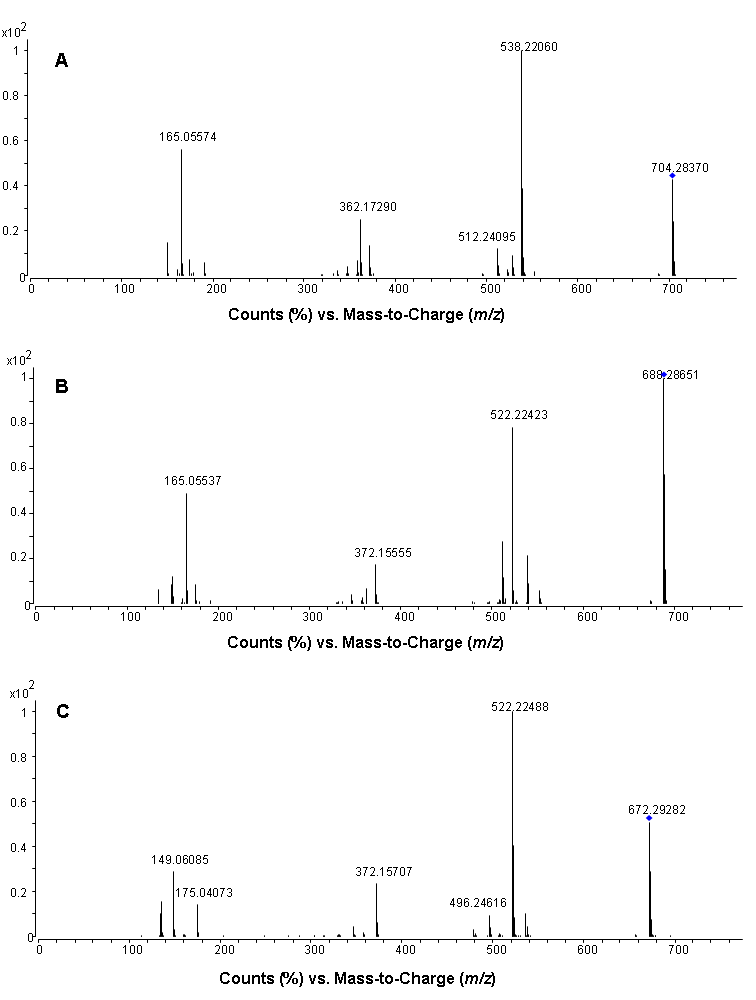


**Additional file 2** (–)ESI-Q-TOF MS/MS spectra of peaks 9 (A), 12 (B) and 14 (C).

Supplement: Supplementary file 2 — Additional file 2:. (−)ESI-Q-TOF MS/MS spectra of peaks 9 (A), 12 (B) and 14 (C). [file 12906_2020_3069_MOESM2_ESM.doc]
