# Supplementary material for: Schisandra chinensis bee pollen’s chemical profiles and protective effect against H2O2-induced apoptosis in H9c2 cardiomyocytes
Source: BMC Complement Med Ther. 2020 Sep 10;20:274. doi: 10.1186/s12906-020-03069-1 (PMC7487998; doi:10.1186/s12906-020-03069-1)
Supplement: Supplementary file 3 — Additional file 3:. The proposed (−)ESI-Q-TOF MS/MS fragmentation pathway of 3–3′-methoxycinnamyl-5-p-coumaroylquinic acid nitrogen-containing derivative. [file 12906_2020_3069_MOESM3_ESM.doc]

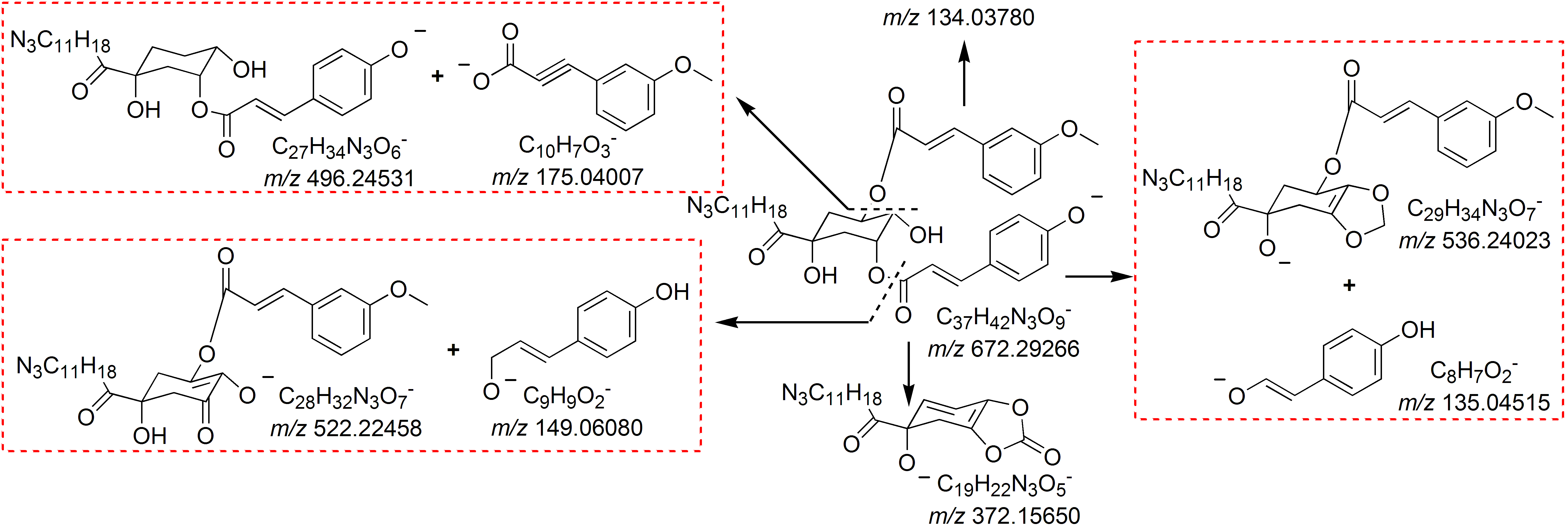


**Additional file 3** The proposed (–)ESI-Q-TOF MS/MS fragmentation pathway of 3-3’-methoxycinnamyl-5-*p*-coumaroylquinic acid nitrogen-containing derivative.
