# Supplementary material for: Schisandra chinensis bee pollen’s chemical profiles and protective effect against H2O2-induced apoptosis in H9c2 cardiomyocytes
Source: BMC Complement Med Ther. 2020 Sep 10;20:274. doi: 10.1186/s12906-020-03069-1 (PMC7487998; doi:10.1186/s12906-020-03069-1)
Supplement: Supplementary file 4 — Additional file 4:. UPLC–DAD Chromatograms at 254 nm of the mixed reference substances including 25 μg/mL of uridine, 26.25 μg/mL of guanosine and 26.75 μg/mL of adenosine (A) and SCBPE (B). [file 12906_2020_3069_MOESM4_ESM.doc]

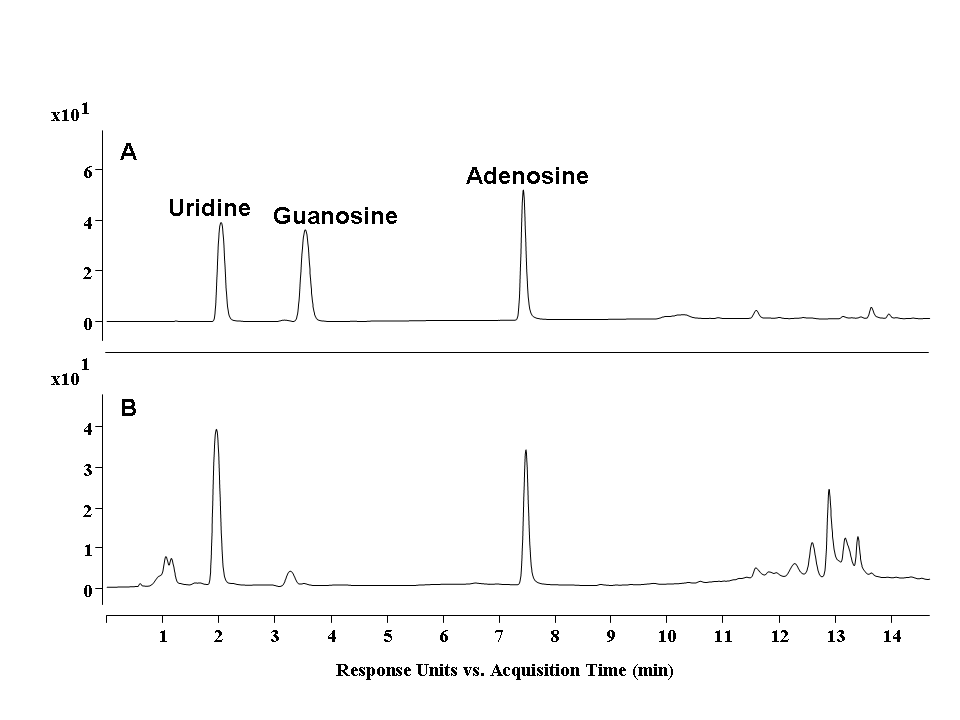


**Additional file 4** UPLC–DAD Chromatograms at 254 nm of the mixed reference substances including 25 µg/mL of uridine, 26.25 µg/mL of guanosine and 26.75 µg/mL of adenosine (A) and SCBPE (B).
