# Supplementary material for: Schisandra chinensis bee pollen’s chemical profiles and protective effect against H2O2-induced apoptosis in H9c2 cardiomyocytes
Source: BMC Complement Med Ther. 2020 Sep 10;20:274. doi: 10.1186/s12906-020-03069-1 (PMC7487998; doi:10.1186/s12906-020-03069-1)
Supplement: Supplementary file 5 — Additional file 5:. Effect of seven concentrations of SCBPE on proliferation of H9c2 cells induced by H2O2 in three time periods, respectively. [file 12906_2020_3069_MOESM5_ESM.doc]

**Additional file 5** Effect of seven concentrations of SCBPE on proliferation of H9c2 cells induced by H2O2 in three time periods, respectively.

| Group | OD value^a^ | | |
| --- | --- | --- | --- |
|  | 24 h | 48 h | 72 h |
| Negative control group | 0.5901±0.0140* | 0.6766 ±0.0124* | 0.7111 ±0.0302* |
| H_2_O_2_ group | 0.0646 ±0.0022^δ^ | 0.0915 ±0.0066^δ^ | 0.4579 ±0.0218^δ^ |
| Positive control (Vc) group | 0.0686 ±0.0022^δ^ | 0.2718 ±0.0136^δ^* | 0.6226 ±0.0102^δ^* |
| 6.25 μg/mL SCBPE | 0.0639 ±0.0007^δ^ | 0.2120 ±0.0117^δ^* | 0.6622 ±0.0153* |
| 12.5 μg/mL SCBPE | 0.0649 ±0.0017^δ^ | 0.2309 ±0.0138^δ^* | 0.6765 ±0.0192* |
| 25 μg/mL SCBPE | 0.0664 ±0.0025^δ^ | 0.2501 ±0.0151^δ^* | 0.7570 ±0.0409* |
| 50 μg/mL SCBPE | 0.0766 ±0.0133^δ^ | 0.2888 ±0.0187^δ^* | 0.6913 ±0.0122* |
| 100 μg/mL SCBPE | 0.0671 ±0.0021^δ^ | 0.2995 ±0.0276^δ^* | 0.7194 ±0.0116* |
| 250 μg/mL SCBPE | 0.0751 ±0.0017^δ^ | 0.3196 ±0.0118^δ^* | 0.5573 ±0.0095^δ^* |
| 500 μg/mL SCBPE | 0.0929 ±0.0055^δ^# | 0.2248 ±0.0106^δ^* | 0.5026 ±0.0108^δ^ |

^a^ Values represent mean ± SD of three independent experiments, and n=8 in each experiment. Compared with negative control group, ^δ^ *P*<0.01; Compared with H_2_O_2_ group, # *p<*0.05, * *p<*0.01.
