# Supplementary material for: Schisandra chinensis bee pollen’s chemical profiles and protective effect against H2O2-induced apoptosis in H9c2 cardiomyocytes
Source: BMC Complement Med Ther. 2020 Sep 10;20:274. doi: 10.1186/s12906-020-03069-1 (PMC7487998; doi:10.1186/s12906-020-03069-1)
Supplement: Supplementary file 6 — Additional file 6: The effect of SCBPE on cell survival rate of H9c2 cells. * Compared with H2O2 group, p < 0.01. # SCBPE+H2O2 groups compared with Vc + H2O2 group, p < 0.05. [file 12906_2020_3069_MOESM6_ESM.doc]

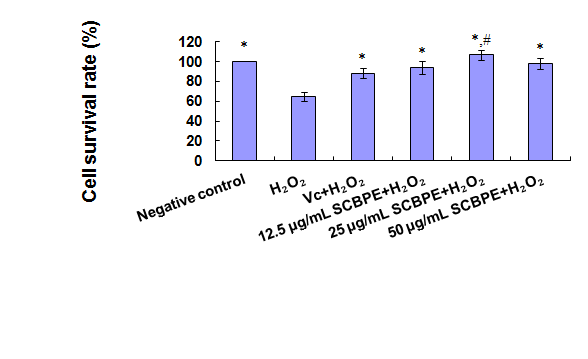


**Additional file 6** Effect of SCBPE on cell survival rate of H9c2 cells. * Compared with H_2_O_2_ group, *p*<0.01. # SCBPE+H_2_O_2_ groups compared with Vc+H_2_O_2_ group, *p*<0.05.
