# Supplementary material for: Schisandra chinensis bee pollen’s chemical profiles and protective effect against H2O2-induced apoptosis in H9c2 cardiomyocytes
Source: BMC Complement Med Ther. 2020 Sep 10;20:274. doi: 10.1186/s12906-020-03069-1 (PMC7487998; doi:10.1186/s12906-020-03069-1)
Supplement: Supplementary file 7 — Additional file 7:. Effect of adenosine, guanosine, uridine and nitrogen-containing quininic acid derivatives on H2O2-induced H9c2 myocardial cell injury, respectively. [file 12906_2020_3069_MOESM7_ESM.doc]

**Additional file 7** Effect of adenosine, guanosine, uridine and nitrogen-containing quininic acid derivatives on H2O2 - induced H9c2 myocardial cell injury, respectively.

| Group | OD value^a^ | | | | | | | | | |
| --- | --- | --- | --- | --- | --- | --- | --- | --- | --- | --- |
|  | Adenosine (n=4) | | Guanosine (n=4) | | Uridine (n=4) | | | Nitrogen-containing quininic acid derivatives (n=6) | | |
|  | 48h | 72h | 48h | 72h | 48h | 72h | | 48h | 72h | |
| Negative control group | 0.6881 ± 0.0061* | 0.9738±0.0497* | 0.7378 ± 0.0072* | 0.9240 ± 0.0159* | 0.4587 ± 0.0077* | | 0.5150 ± 0.0084* | 0.4452 ± 0.0136 * | | 0.5210 ± 0.0027 * |
| H_2_O_2_ group | 0.6036 ± 0.0058 | 0.8816 ± 0.0069 | 0.6319 ± 0.0195 | 0.8111 ± 0.0041 | 0.2892 ± 0.0089 | | 0.3294 ± 0.0095 | 0.1580 ± 0.0047 | | 0.3371 ± 0.0440 |
| 0.2 μg/mL | 0.7512 ± 0.0044* | 0.9259 ± 0.0092 | 0.6771 ± 0.0127* | 0.8643 ± 0.0542 | ̵ ^b^ | | ̵ ^b^ | 0.1960 ± 0.0125 * | | 0.4578 ± 0.0318 * |
| 0.39 μg/mL | 0.7677 ± 0.0095* | 0.9294 ± 0.0130 | 0.6816 ±0.0099* | 0.9123 ± 0.0053* | ̵ ^b^ | | ̵ ^b^ | 0.2096 ± 0.0109 * | | 0.4601 ± 0.0073 * |
| 0.78 μg/mL | 0.7173 ± 0.0091* | 0.9565 ± 0.0276# | 0.6853 ± 0.0059* | 0.8379 ± 0.0064 | 0.3052 ± 0.0038 | | 0.3596 ± 0.0061 | 0.2125 ± 0.0132 * | | 0.5151 ± 0.0347 * |
| 1.56 μg/mL | 0.7156 ± 0.0091* | 0.9837 ± 0.0073* | 0.6865 ± 0.0065* | 0.9120 ± 0.0341* | 0.3068 ± 0.0086 | | 0.3672 ± 0.0036 | 0.2370 ± 0.0119 * | | 0.5968 ± 0.0127 * |
| 3.125 μg/mL | 0.7437 ± 0.0094* | 0.9758 ± 0.0124* | 0.6842 ± 0.0156* | 0.7989 ± 0.0386 | 0.3081 ± 0.0104 | | 0.3504 ± 0.0108 | 0.2079 ± 0.0052 * | | 0.5864 ± 0.0106 * |
| 6.25 μg/mL | 0.7599 ± 0.0096* | 0.9372 ± 0.0162 | 0.6577 ±0.0109 | 0.8472 ± 0.0181 | 0.3109 ±0.0052 | | 0.3494 ± 0.0041 | 0.1869 ± 0.0069 # | | 0.5220 ± 0.0302 * |
| 12.5 μg/mL | 0.7373 ± 0.0107* | 0.9691 ± 0.0097* | 0.6257 ± 0.0063 | 0.7788 ± 0.0097 | 0.3129 ± 0.0024 | | 0.3665 ± 0.0023 | 0.1895 ± 0.0018 # | | 0.3996 ± 0.0136 |
| 25 μg/mL | 0.7577 ± 0.0016* | 1.0599 ± 0.0239* | 0.5985 ± 0.0072# | 0.8381 ± 0.0110 | 0.3166 ± 0.0014 | | 0.3862 ± 0.0030# | 0.1171 ± 0.0040 * | | 0.2273 ± 0.0164 * |
| 50 μg/mL | 0.7660 ± 0.0105* | 0.9217 ± 0.0030 | 0.5177 ± 0.0051* | 0.7780 ± 0.0051 | 0.3499 ± 0.0100 | | 0.4123 ± 0.0064* | ̵ ^b^ | | ̵ ^b^ |
| 100 μg/mL | 0.6622 ±0.0193* | 0.8801 ± 0.0053 | 0.1822 ± 0.0134* | 0.6144 ± 0.0131* | 0.3755 ± 0.0157* | | 0.3970 ± 0.0030* | ̵ ^b^ | | ̵ ^b^ |

^a^ Values represent mean ± SD. ^b^ This concentration of the component has not been detected. Compared with H_2_O_2_ group, # *p<*0.05, * *p<*0.01.
